# Associations of Polymorphisms in WNT9B and PBX1 with Mayer-Rokitansky-Küster-Hauser Syndrome in Chinese Han

**DOI:** 10.1371/journal.pone.0130202

**Published:** 2015-06-15

**Authors:** Wenqing Ma, Ya Li, Man Wang, Haixia Li, Tiefen Su, Yan Li, Shixuan Wang

**Affiliations:** 1 Department of Gynecology and Obstetrics, Tongji Hospital, Tongji Medical College, Huazhong University of Science and Technology, Wuhan, Hubei, P.R. China; 2 Department of Pathology, Tongji Hospital, Tongji Medical College, Huazhong University of Science and Technology, Wuhan, Hubei, P.R. China; Institut Jacques Monod, FRANCE

## Abstract

**Background:**

Mayer-Rokitansky-Küster-Hauser (MRKH) syndrome is a rare syndrome that is characterized by congenital aplasia of the uterus and the upper portion (2/3) of the vagina. Previous attempts to identify causal mutations of MRKH syndrome have primarily resulted in negative outcomes. We investigated whether these reported variants are associated with MRKH syndrome (types I and II) in a relatively large sample size of Chinese Han patients, and whether any gene-gene epistatic interactions exist among these variants.

**Methods:**

This study included 182 unrelated Chinese women with MRKH syndrome (155 with type I and 27 with type II) and 228 randomized female controls. Seventeen candidate loci in the AMH, PBX1, WNT4, WNT7A, WNT9B, HOXA10, HOXA11, LHXA1 and GALT genes were genotyped using the Sequenom MassARRAY iPLEX platform. Single-marker association, additive effects and multifactor interactions were investigated.

**Results:**

The gene frequency distributions of MRKH type 1 and type 2 were similar. Rs34072914 in WNT9B was found to be associated with MRKH syndrome (P = 0.024, OR = 2.65, 95%CI = 1.14–6.17). The dominant models of rs34072914 and rs2275558 in WNT9B and PBX1, respectively, were significantly associated with MRKH syndrome risk in the Chinese Han patients. Additive gene-gene interaction analyses indicated a significant synergetic interaction between WNT9B and PBX1 (RERI = 1.397, AP = 0.493, SI = 4.204). Multifactor dimensionality reduction (MDR) analysis revealed novel dimensional epistatic four-gene effects (AMH, PBX1, WNT7A and WNT9B) in MRKH syndrome.

**Conclusions:**

This association study successfully identified two susceptibility SNPs (WNT9B and PBX1) associated with MRKH syndrome risk, both separately and interactively. The discovery of a four-gene epistatic effect (AMH, PBX1, WNT7A and WNT9B) in MRKH syndrome provides novel information for the elucidation of the genetic mechanism underlying the etiology of MRKH syndrome.

## Introduction

Mayer-Rokitansky-Küster-Hauser (MRKH) syndrome (MIM 277000) is a congenital malformation of the female reproductive system that is characterized by agenesis of the Müllerian structures. The principal feature of MRKH syndrome is the absence of the uterus and the upper portion (2/3) of the vagina. Patients normally carry a 46,XX karyotype, identifiable secondary sexual characteristics and functioning ovaries with no signs of androgen excess. Associated malformations (MRKH type II) include renal abnormalities (unilateral agenesis, renal ectopia and horseshoe kidney), skeletal malformations (Klippel-Feil anomaly, fused vertebrae, mainly cervical and scoliosis), and more rarely, cardiac and digital anomalies (syndactyly and polydactyly) [1]. The congenital malformations affect 1 in every 4,500 female live births and cause incurable infertility and sexual dysfunction [2]. Thus, over 810,420 patients are mentally and physically suffering from this type of infertility worldwide.

The etiology of MRKH syndrome remains obscure [3]. It was initially observed sporadically, suggesting the involvement of environmental factors, such as thalidomide-like teratogens or diabetes [4]. However, no association with illness, drug use, or teratogen exposure has been observed in studies of available pregnancy histories [5]. The Müllerian ducts (MD) form as an invagination of the coelomic epithelium during embryonic development, extend caudally through epithelialization along the Wolffian ducts (WD), and finally grow into the Fallopian tubes, the uterus and the upper two-thirds of the vagina [6]. Numerous genes exhibiting a wide range of activity during MD development, such as PBX1, HOX family genes, WNT family genes and ant-Müllerian hormone (AMH), have been suggested as candidates for MRKH syndrome based on phenotypes that have been observed in mutant mice [7]. The roles of these genes in this syndrome were preliminarily examined using Sanger sequencing. Table 1 lists the candidate genes, and S1 Table details their reported genotype frequencies.

**Table 1:** 17 candidate loci in genes involved in Müllerian duct development.

Gene	Knockout	Locus	Location		Reference
***Formation***					
LHX1	No MD	Novel, Mis	c.791G>C, p.264R>P	Ledig, S et al., 2011	
WNT4	No MD, only MD	Novel, Mis	c.35T>C, p.12L>P	Philibert, P et al., 2008	
	precursor cells with	rs16826648	c.276G>A, p.92L	Chang, X et al., 2012	
	Lhx1 expression	Novel, Mis	c.697G>A, p.233A>T	Philibert, P et al., 2011	
		Novel, Syn	c.483C>T, p.161Y	Ravel, C et al., 2009	
WNT9B	Invagination of MD,	rs4968281, Mis	c.317T>C, p.106 M >T	Ravel, C et al., 2009	
	no elongation along WD	rs34072914, Syn	c.399G>T, p.133R	Wang, M et al., 2014	
		Novel, UTR3	c.*158 C>T	Wang, M et al., 2014	
***Differentiation***					
WNT7A	MD formed, no proper	rs3749319, Intron	c.298+37C>A		Dang, Y et al., 2012
	differentiation of	Novel, Syn	c.342C>T, p.114G		Dang, Y et al., 2012
	oviduct and uterus	rs3762719,Syn	c.459T>C, p.153S		Dang, Y et al., 2012
	tissue	Novel, Syn	c.861G>A, p.287V		Ravel, C et al., 2009
HOXA10	the anterior part of the	Novel, Mis	c.170A>G, p.57Y>C		Cheng, Z et al., 2011
	uterus is transformed				
	into the oviduct				
HOXA11	Abnormal FRT	Novel, Mis	c.113C>G, p.38P>R		Liatsikos, SA et al., 2012
PBX1	No MD	rs2275558, Mis	c.61G>A, p.21G>S		Ma, J et al., 2011
***Regression***					
AMH	Persistent MD in males	Novel, Mis	c.934C>T, p.312R>C		Zenteno, JC et al., 2004
GALT		rs2070074, Mis	c.940A>G, p.314N>D		Zenteno, JC et al., 2004

MD, Müllerian duct; WD, Wolffian duct; FRT, female reproductive tracts; Syn, Synonymous mutations; Mis, Missense mutations; UTR3, the 3’ untranslated region.

Table 1 and S1 Table show that some novel single nucleotide variants (SNVs) and existing single nucleotide polymorphisms (SNPs) have been detected in these candidate genes. However, these results require further validation and replication in larger cohorts of different races for the reasons discussed below [6, 8–18]. First, alleles at different loci that cause a particular disease are heterogeneous in different populations because of inherent bias. Therefore, re-evaluations of these SNVs in MRKH patients and their potential associations with this syndrome in different populations would be meaningful [19]. Second, the sample sizes of previous studies did not generate enough power to detect pathogenic variants with only mild effects [20, 21]. Third, interactions of multiple-genes or-factors must be highlighted in a possibly complex disorder, such as MRKH syndrome. Finally and most importantly, the phenotypes of the previously studied cases have not been pure because the studies have included patients with other reproductive tract malformations, such as unicornuate uterus, septate uterus and vaginal septum [10, 11, 13]. MD malformations include a variety of congenital anomalies that result from the incomplete fusion of mesonephric ducts, abnormal formation or arrested development [22]. It is impossible to identify the pathogenic mutations of MD anomaly (MDA) because of the wide range of phenotypes. However, studies of MRKH syndrome, which is the most common and serious type of MDA [23], and its etiological mechanisms may provide the critical key for unlocking the mystery of MDAs. Therefore, etiological studies of large numbers of MRKH syndrome patients of different races are urgently needed.

We investigated the associations between MRKH syndrome and 17 candidate gene loci reported in eligible published studies (Table 1) in 182 MRKH syndrome patients (155 type I and 27 type II) and 228 female controls of Han Chinese ethnicity to verify and replicate these candidate variants in a Chinese population. In addition, we searched for associations of MRKH syndrome with combinations of two- and multiple-loci using new statistical approaches to examine the multifactor interactions involved in etiology.

## Materials and Methods

### Study populations

A cohort of 182 unrelated Han females with MRKH syndrome (age 25.30±5.02 years) were recruited from the central and southern regions of China from 2011–2012. Diagnoses of all patients were based on comprehensive gynecological examination performed by 3 gynecologists (Man Wang, Ya Li and Yan Li), which included obtaining a medical history and conducting physical examination, hormone analysis, karyotype analysis, pelvic and abdominal ultrasonography and laparoscopy or surgery (details listed in S2–S4 Tables). All patients had normal external genitalia and ovaries, and the karyotype of all tested patients was 46,XX. The results of blood tests revealed normal levels of estradiol, FSH, LH and testosterone, and none of these women had a history of diethylstilbestrol exposure or diabetes or evidence of hyperandrogenism. The integration of medical history, general physical examination and inspection revealed that these patients had associated malformations, including renal anomaly (17/182), skeletal defect (4/182), both renal and skeletal malformations (1/182), hernia (3/182), atrial septal defect (1/182) and schizophrenia(1/182).

The control group consisted of 228 region-matched women, who had at least one normal pregnancy with eutocia. The ethnic background of the participants in this study was Chinese Han. The Medical Ethics Committee of the Tongji Medical College, Huazhong University of Science and Technology approved this study, and all participants provided written informed consent for sample collection and subsequent analysis.

### Candidate loci selection and genotyping

A thorough search of PubMed was performed to select reported SNVs related to MRKH syndrome. The search included all papers published until September 31, 2013, using a combination of the following key words: "congenital absence of the vagina", "MRKH", "Mayer-Rokitansky-Küster-Hauser syndrome", "CAUV", "MURCS", "Müllerian aplasia", "Müllerian duct abnormalities", "MDAs" and "polymorphism", "genotype", and "allele". The references of the retrieved papers were also screened for suitable papers. The following criteria were adopted for selection: (1) case-control studies that provided MRKH syndrome diagnoses, using subjects without female reproductive tract defects as controls; (2) papers that listed sample sizes and the genotype frequency for each variant; (3) studies concerning the mutations of candidate genes and the risk of MRKH syndrome; and (4) studies in which the reported candidate loci were verified by performing GenBank searches. A total of 17 SNVs in MRKH syndrome candidate genes were included in this study, the details of which are listed in S1 Table.

Total genomic DNA was extracted from 200 μL peripheral blood using a Fuji Film Quick Gene DNA whole blood kit S, according to the manufacturer's instructions. DNA samples were amplified by multiplex PCR, and the amplification products were used for locus-specific single-base extension reactions. Genotyping analysis of the selected SNVs was performed using Sequenom MassARRAY technology (San Diego, California, USA). Locus-specific PCR and detection primers were designed using MassARRAY Assay Design 3.0 software (S5 Table). Allele detection was performed using matrix-assisted laser desorption/ionization time-of-flight mass spectrometry (Sequenom). Mass spectrograms were analyzed using MassARRAY Typer software. Detected loci with call rate of lower than 95% in the cases and controls were excluded.

### Statistical analysis

Fisher's exact test was used to test Hardy-Weinberg equilibrium (HWE) for each SNP in the control subjects. The χ^2^-test was used to compare the genotype frequency distributions between the cases and controls. All odds ratios were calculated for the minor allele of each variant. All P-values were two-tailed and an alpha of 0.05 was used to determine statistical significance.

Sorting Intolerant From Tolerant (SIFT) (http://sift.bii.a-star.edu.sg/www/SIFT_BLink_submit.html), PolyPhen-2 (http://genetics.bwh.harvard.edu/pph2) and PMut (http://mmb2.pcb.ub.es:8080/PMut) were used in this study to predict whether an amino acid substitution in a protein exerted a phenotypic effect. Alignments of amino acid and nucleic acid sequences of 8 species (including *Homo sapiens*, *Pan troglodytes*, *Mus musculus*, *Bos taurus*, *Felis catus*, *Sarcophilus harrisii*, *Gallus gallus* and *Danio rerio*) were performed using Clustal Omega-Multiple Sequence Alignment (http://www.ebi.ac.uk/Tools/msa/clustalo/).

We also explored interactive effects among candidate loci for MRKH syndrome. We performed the following three measurements in additive gene interaction analyses [24]: relative excess risk due to interaction (RERI); the attributable proportion due to interaction (AP); and the synergy index (SI). High dimensional multifactor interactions were assessed using multifactor dimensionality reduction (MDR; http://sourceforge.net/projects/mdr/) software. All possible interactions of the qualified SNVs were tested using the default MDR parameters in an exhaustive search.

## Results

### Single SNP association study

As showed in Table 2 and S6 Table, The genotype frequency distributions of MRKH types I and II were basically consistent. All SNVs in the control subjects were under HWE, except for the novel variant c.35T>C in exon 1 of WNT4 and rs4968281 in WNT9B (S6 Table). Among the remaining 15 SNVs, rs34072914 in WNT9B was significantly associated with MRKH syndrome in this Chinese cohort (P = 0.026, OR = 2.575, 95%CI = 1.089–6.085). This SNV resulted in a synonymous substitution of guanine with thymine at nucleotide position 399 in exon3 (c.399G>T), which did not change the amino acid sequence. However, patients with the minor T allele showed an increased risk of MRKH syndrome, including both types I and II (Table 2). This association was also identified for MRKH type II (P = 0.010, OR = 4.480, 95%CI = 1.303–15.408), in a relatively small sample size of 27 patients. The variant c.934C>T in AMH was significantly associated with isolated MRKH syndrome (P = 0.049 and OR = 0.346, 95%CI = 0.115–1.043), and it resulted in the change of arginine to cysteine at residue 312. A protective effect of this variant was consistently observed in the patients with MRKH type II. This mutation was predicted to be likely damaging using PolyPhen-2, with a score of 1.00 (specificity: 1.00), as well as by PMut, with an Output of 0.86 (reliability: 7).

**Table 2:** Association of single nucleotide polymorphisms with risk of MRKH syndrome in Chinese population.

				MRKH type I		MRKH type II		MRKH	
Chr	Position	Gene	SNP	OR(95%CI)	P	OR(95%CI)	P	OR(95%CI)	P
1	164529120	PBX1	rs2275558	1.109(0.825–1.490)	0.492	1.401(0.772–2.542)	0.266	1.147(0.865–1.521)	0.341
3	13896140	WNT7A	rs3762719	0.813(0.609–1.086)	0.161	1.295(0.727–2.307)	0.379	0.871(0.660–1.147)	0.325
3	13916407	WNT7A	rs3749319	0.894(0.638–1.254)	0.516	0.768(0.407–1.449)	0.414	0.874(0.633–1.205)	0.410
7	27216334	HOXA10	c.170A>G	NA	0.276	0.344(0.035–3.367)	0.356	0.415(0.043–4.012)	0.780
9	34649442	GALT	rs2070074	1.104(0.245–4.968)	1.000	NA	1.000	0.939(0.209–4.223)	1.000
17	44952531	WNT9B	rs34072914	2.255(0.911–5.583)	0.071	**4.480(1.303–15.408)**	**0.010**	**2.575(1.089–6.085)**	**0.026**
17	44959243	WNT9B	c.*158 C>T	NA	0.405	NA	NA	NA	0.444
19	2251207	AMH	AMH c.934C>T	**0.346(0.115–1.043)**	**0.049**	0.565(0.065–4.935)	0.479	0.368(0.125–1.087)	0.060

Co-dominant, dominant and recessive models for each SNV were also tested (S7 Table), and the positive results are listed in Table 3. The frequency of the dominant model (*TG/TT*) of rs34072914 in WNT9B was significantly increased in the women with MRKH syndrome compared with the controls (P = 0.039, OR = 2.47, 95%CI = 1.02–5.96), and this result was consistently observed in the women with MRKH type II (P = 0.027, OR = 4.78, 95%CI = 1.34–17.11). A harmful effect was also detected for the dominant model (*AG/AA*) of rs2275558 in PBX1 (P = 0.046, OR = 1.749, 95%CI = 1.01–3.04). This SNP causes a substitution from glycine to serine at position 21 of the Pbx1 amino acid sequence. SIFT and PMut programs predicted that the SNP rs2275558 was unlikely to have functional consequences, but PolyPhen-2 predicted that it was likely damaging, with a score of 0.491 (sensitivity: 0.89; specificity: 0.90). A separate genetic model analysis of MRKH type I demonstrated that the recessive model (*TT*) of AMH c.934C>T was associated with MRKH type I (P = 0.047, OR = 0.34, 95%CI = 0.11–1.03). There were no significant differences among the frequencies of the other 12 SNVs between the patients and normal females.

**Table 3:** Genetic Model.

Phenotype	Gene	Genotype	Case	Control	OR(95%CI)	P
MRKHS	WNT9B	Dominant				
	rs34072914	TG/TT	15	8	2.47(1.02–5.96)	0.039
		GG	167	220		
MRKHS	PBX1	Dominant				
	rs2275558	AA/AG	160	183	1.749(1.01–3.04)	0.046
		GG	22	44		
Type II	WNT9B	Dominant				
	rs34072914	TG/TT	4	8	4.78(1.34–17.11)	0.027
		GG	23	220		
Type I	AMH	Recessive				
	c.934C>T	TT	136	223	0.34(0.11–1.03)	0.047
		TC/CC	9	5		

**Fig 1:**
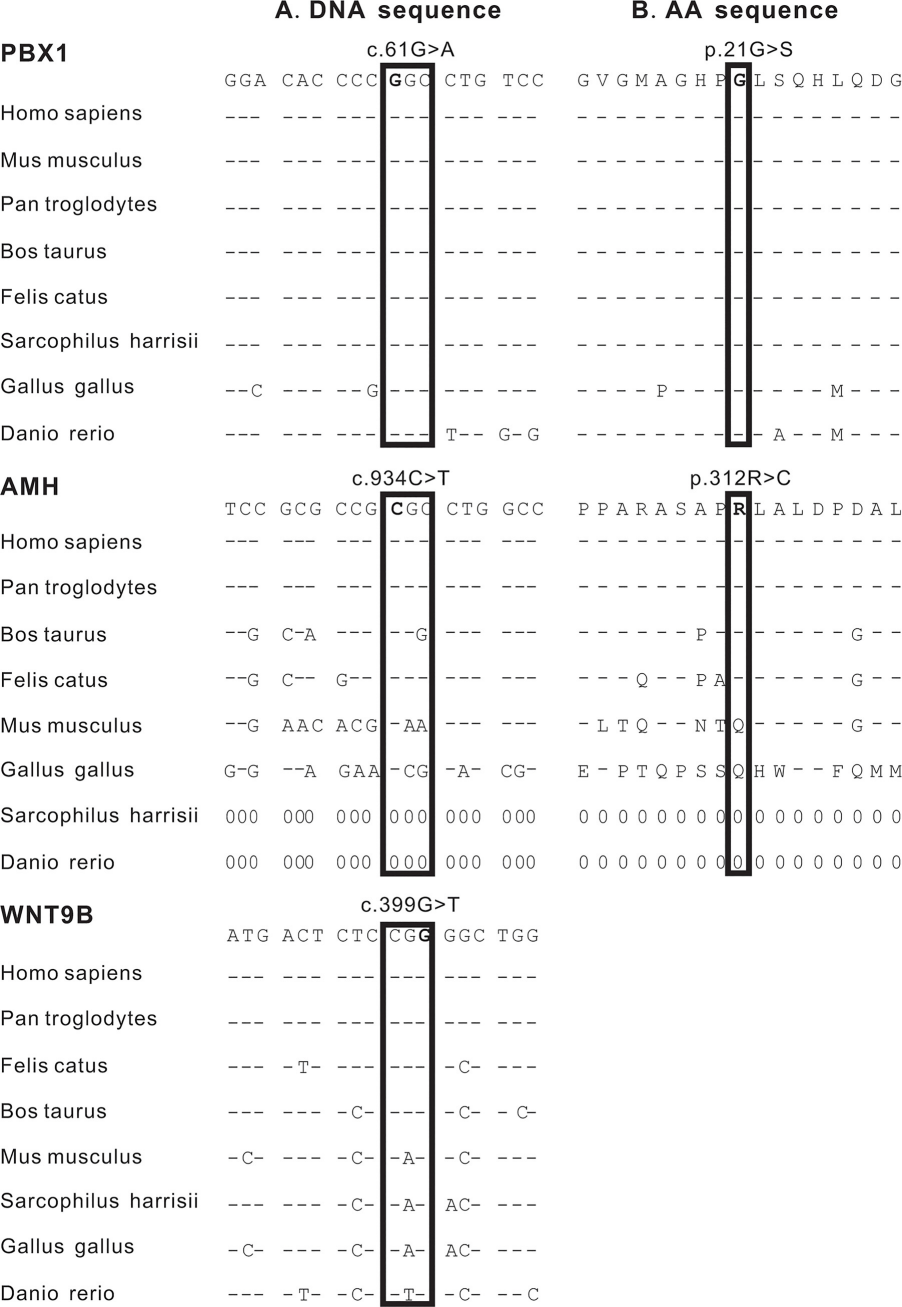
Sequence alignments of wild-type rs2275558, c.934C>T in AMH and rs34072914 of eight species. **(A)** Nucleic acid sequence alignments of rs227558 in PBX1, c.934C>T in AMH and rs34072914 in WNT9B of eight different species. (**B)** Amino acid sequence alignment of PBX1 p.21G>S and AMH p.312R>C of eight different species. ClustalW program was used to perform alignments. The bold letters indicate the 3 variants of interest. The hyphen indicates that the nucleotide base or amino acid is consistent with that in the human wild-type gene or protein, respectively. Zero means there is no corresponding sequences. Rs2275558 in PBX1 was consistently present in all 8 vertebrates, and rs34072914 in WNT9B and c.934C>T in AMH presented relatively conservative in mammals.

The conservation of amino acid or nucleic acid sequences may reflect their importance to some extent. Sequences alignments of 8 species (including *Homo sapiens*, *Pan troglodytes*, *Mus musculus*, *Bos taurus*, *Felis catus*, *Sarcophilus harrisii*, *Gallus gallus* and *Danio rerio*) revealed that rs2275558 in PBX1 was consistently present in all 8 vertebrates, and rs34072914 in WNT9B and c.934C>T in AMH presented relatively conservative in mammals (Fig 1).

### Additive gene-gene interaction analysis

We explored the possible additive interactions between pairs of candidate loci for MRKH syndrome by calculating the RERI, AP and SI (S8 Table). These measurements indicate significant interactions when they differ from 0 (RERI and AP) or 1 (SI). A significant synergistic additive interaction was observed between rs34072914 *GT+TT* in WNT9B and rs2275558 *GA+AA* in PBX1 (P = 0.032, OR = 2.833, 95%CI = 1.055–7.609), with an RERI of 1.397, AP of 0.493 and SI of 4.204. A similar tendency was also observed between rs34072914 *GT+TT* in WNT9B and rs3749319 *CA+AA* in WNT7A (P = 0.026, OR = 3.148, 95%CI = 1.088–9.107, RERI = 2.211, AP = 0.702) and rs34072914 *GT+TT* in WNT9B and rs3762719 *TC+CC* in WNT7A (P = 0.026, OR = 3.148, 95%CI = 1.088–9.107, RERI = 2.211, AP = 0.702).

### High-dimensional MDR interaction analysis

**Fig 2:**
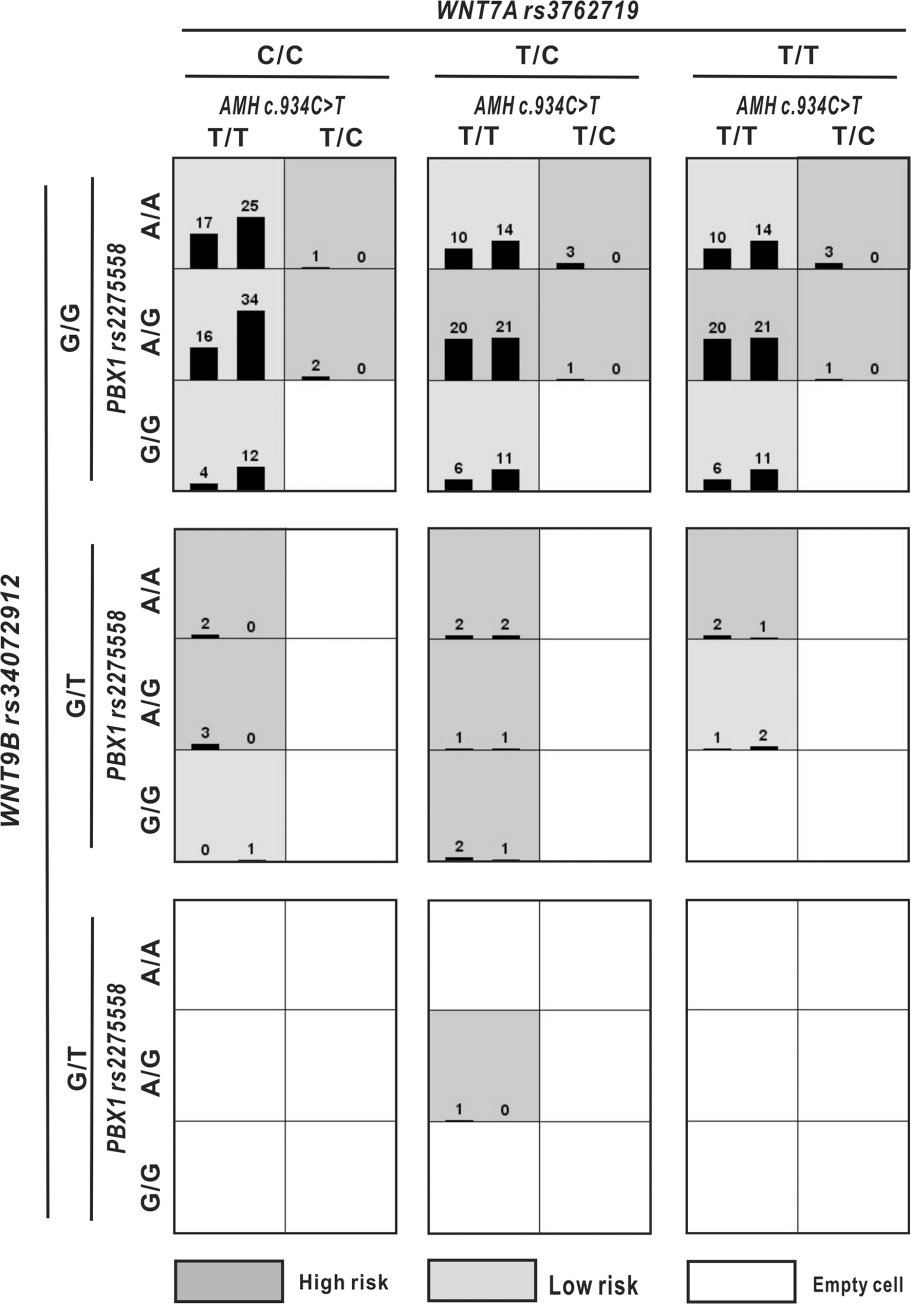
The optimal models as determined by MDR analysis for AMH c.934C>T, PBX1 rs2275558, WNT7A rs3762719 and WNT9B rs34072914. Summary of four-locus genotype combinations associated with a high risk and low risk of MRKH syndrome, with the corresponding distribution of cases (left bars in boxes) and of controls (right bars in boxes), for each multi-locus-genotype combination. The four-locus model (AMH c.934C>T, PBX1 rs2275558, WNT7A rs3762719 and WNT9B rs34072914) was found to result in an increased the risk of MRKH syndrome (OR = 2.268, 95%CI = 1.494–3.443).

We identified potential high-dimensional gene-gene interactions by MDR analysis. The two- to four-way models used are listed in Table 4. The two-way model with PBX1 rs2275558 and WNT7 rs3762719 showed the highest testing-balanced accuracy. A three-way interaction was found among PBX1 rs2275558, WNT7 rs3762719 and WNT9B rs34072914. A four-locus model (AMH c.934C>T, PBX1 rs2275558, WNT7 rs3762719 and WNT9B rs34072914) was chosen as the appropriate model, showing the highest testing-balanced accuracy of 59.61% and the highest cross-validation consistency of 10/10. The four-locus model was found to result in an increased the risk of MRKH syndrome (OR = 2.268, 95%CI = 1.494–3.443). Details of the high-risk and low-risk models are shown in Table 4 and Fig 2.

**Table 4:** Gene-gene interaction test.

Model	Training Bal. Acc. (%)	Testing Bal. Acc. (%)	Cross-validation Consistency
(PBX1)rs2275558/(WNT7)rs3762719	0.5679	0.5354	9/10
(PBX1)rs2275558/(WNT7)rs3762719 /(WNT9B) rs34072914	0.5870	0.5497	10/10
AMH c.934C >T/ (PBX1)rs2275558/(WNT7A)rs3762719 /(WNT9B) rs34072914	0.5981	0.5617	10/10

## Discussion

MRKH syndrome causes a loss of sexuality and fertility in patients, and effective treatments for this disorder are lacking. A deeper understanding of its etiology would facilitate early diagnosis and effective intervention and preventive measures. However, geneticists have struggled for decades to define a variant that causes MRKHS, obtaining negative results. Increasing knowledge about genetic diseases has prompted the current definition of MRKH syndrome as a syndrome of polygenic or multifactorial origin, with each factor explaining only a small fraction of its overall heritability. Genetic association studies offer a potentially powerful approach for the mapping of causal genes with modest effects [21]. Therefore, we conducted this association study using Chinese Han patients to verify the contribution of candidate gene loci to MRKH syndrome as well as their potential interactions.

Several related genes were screened in MRKH syndrome patients based on the findings of previous studies of the mechanism of MD development, and candidate SNVs were detected. However, causal mutations were not identified in any of these genes, partially because of a lack of validation in large case-control cohorts. The present genotyping study of a relatively large number of MRKH patients verified 17 candidate loci in the PBX1, WNT4, WNT7A, WNT9B, HOXA10, HOXA11, GALT, LHXA1 and AMH genes. With the exception of rs34072914, rs2275558, c.934C>T in AMH and 2 SNPs that were not in HWE, the remaining 12 loci were not found to be associated with MRKH syndrome type I or type II in our cohort (S6 Table). We failed to replicate the 7 following previously reported variants: rs16826648 [10], c.697G>A [15] and c.483C>T [8] in WNT4; c.342C>T [10] and c.861G>A [8] in WNT7A; c.113C>G [18] in HOXA11; and c.791G>C [25] in LHX1. The remaining 5 variants (rs3749319 [10] and rs3762719 [10] in WNT7A; C.170A>G [17] in HOXA10; rs2070072 [14] in GALT; and c.*158C>T [16] in WNT9B) were detected, but there were no significant differences in the frequencies of these genotypes or alleles between the cases and controls in this Chinese cohort (Table 2). These discrepancies may be due to differences among the study populations assessed and genetic heterogeneity, which has been observed in previous studies, especially in studies of monozygotic twins [26, 27]. The inconsistent results of candidate gene studies and the reported discordance in monozygotic twins suggest the presence of wide genetic heterogeneity among MRKH syndrome patients. The findings of this study suggest that these 12 variants are not associated with the pathogenesis of MRKH syndrome in the Han population. However, we cannot eliminate their possible role as putative MRKH genes, and their pathogenicities in other ethnicities require further study in large samples of patients from specific populations.

We have identified the SNP rs34072914 is associated with MRKH syndrome in Chinese Han patients. Furthermore, the dominant model (*TG/TT*) of rs34072914 significantly increased the risk of this syndrome (P = 0.039, OR = 2.47, 95%CI = 1.02–5.96). These associations were particularly evident for MRKH type II, and MRKH type I showed the same tendency. The conservation of this nucleotide locus across mammalian species also verifies its importance to the normal functioning of the WNT9B gene. This gene is a member of the WNT family, which is a group of structurally related genes that act as extracellular signaling factors [28]. A study of Wnt9b -/- mice has demonstrated that Wnt9b is essential for the development of mesonephric and metanephric tubules and the caudal extension of the MD [29]. Further analysis has shown that Wnt9b is required for the earliest inductive response in the metanephric mesenchyme [29]. The SNP rs34072914 causes no change in the WNT9B amino acid sequence, but a growing body of evidence has revealed that synonymous SNPs perturb cellular functions and cause distinct clinical phenotypes through several mechanisms, such as affecting messenger RNA splicing, stability and structure as well as protein folding [30]. The results obtained from our cohort suggest that rs34072914 interferes with the normal functioning of WNT9B and other related genes or pathways, perturbs MD development, causing the MRKH syndrome phenotype or multiple system malformations. Functional studies and further research using larger cohorts of patients from other ethnicities are needed to further test this hypothesis.

PBX1 encodes a three amino acid loop extension (TALE) class homeodomain protein that participates in multimeric transcriptional complexes to regulate gene expression during development [31]. Pbx1 is widely expressed in mesenchymal tissues, and its loss markedly reduces outgrowth of thee urogenital ridge and leads to impaired differentiation of the mesonephros and kidneys. The inactivation of PBX1 in the mammalian female reproductive system leads to the absence of Müllerian structures. PBX1 is likely involved in MRKH syndrome [32]. Recently, mutations in 9 exons and exon-intron boundaries of the PBX1 gene were screened in a cohort of patients with MDA in the absence of a control group, and two SNPs were identified [13]. One of these SNPs was rs2275558, with genotype distributions of *GG* 38%, *GA* 32% and *AA* 30% in the patients with MDAs, which obviously differ from the genotyping results of this study (*GG* 12.2%, *GA* 51.6%, *AA* 36.3% in the MRKH patients and *GG* 19.3%, *GA* 43.4%, *AA* 36.8% in the matched control group). These discordant results may be due to diverse phenotypes of patients with MDA (only 9.9% have MRKHS), the majority of whom have a malformed uterus, as well as inherent bias between the populations of northern and southern China, which is also apparent in Database 1000 Genomes(http://browser.1000genomes.org). The dominant model (*AG/AA*) of PBX1 rs2275558 was found to be significantly associated with MRKH syndrome in this study, and made the genotype carriers 1.75 times higher risk of MRKH syndrome. This missense SNP was predicted to cause possible phenotypic effects, and the high conservation of this amino acid residue in mammals indicates its indispensable value for the normal functioning of the PBX1 gene. It is necessary to study PBX1 variants more extensively to clarify the precise molecular mechanism.

We also investigated the genotype differences between MRKH type I and type II separately. Genotyping and statistical analysis reveal that the genotype frequency distributions of the 17 candidate loci were similar between MRKH type I and type II. We also demonstrated that the variant c.934C>T (p.312R>C) of AMH was associated with MRHK type I and played a protective role. This same tendency was also found for MRKH type II. AMH signal transduction induces the degradation of MDs, and has been long implicated in MRKH syndrome. Previous studies have screened the entire AMH gene and its receptor for DNA sequence variations and have measured this hormone levels in MRKH syndrome patients, reporting negative results [14, 33, 34]. The associated mutation c.934C>T, particularly the homozygous mutation (TT), was predicted to interfere with the normal functioning of the AMH protein and to disturb AMH-dependent Müllerian degeneration. Therefore, individuals with the T allele at c. 934 of AMH may have only a one-third risk of MRKHS. This finding highlighted the participation of AMH signaling in the etiology of MRKH syndrome and suggested us its functional mechanism.

Gene-gene interaction analysis indicated tendencies toward multiple interactions among thee above genes. We observed significant additive interaction between the variants rs34072914 in WNT9B and rs2275558 in PBX1, which were each separately associated with MRKH syndrome risk in this cohort. These genes acted synergistically (RERI = 1.397, SI = 4.204) to increase the risk of MRKH syndrome. Among the individuals carrying these variant alleles, 49.3% (AP = 0.493) of MRKH syndrome risk was attributable to the interaction of both variants compared with the individual contribution of each of the two risk factors added together. The biological interaction between WNT9B and PBX1 has been studied in cleft lip and/or palate resulting from abnormal morphogenesis of the face [35]. In mouse lines lacking the Pbx gene in the cephalic ectoderm, the Pbx protein has been shown to directly regulate Wnt signaling by binding to a Wnt9b-Wnt3 mid-facial regulatory element. Moreover, Pbx-regulated Wnt signaling is essential for mid-facial morphogenesis. *Wnt9b* is also expressed throughout the WD (rather than MD) epithelium during urogenital development [29]. The WD delivers signals to the MD to extend caudally through epithelialization from MD mesoepithelium [6]. Wnt9b is a paracrine factor, and it is plausible that this signaling molecule activates the canonical Wnt signaling process during urogenital development [29]. Based on these findings, we propose that Wnt9b in the WD and Pbx1 in the mesoepithelium may directly and indirectly interact during elongation of the MD to activate Wnt signaling, induce epithelialization of the MD mesoepithelium to form the MD and guide it to extend caudally along the WD.

Our study also demonstrated a tendency for gene-gene interactions between WNT9B rs34072914 and WNT7A rs3749319, as well as WNT9B rs34072914 and WNT7A rs3762719. WNT7A belongs to the WNT family, and it is expressed as a regulatory factor in the MD [36]. Wnt7a expression in Wnt9b mutants is restricted to an anterior epithelial structure that corresponds to the initial coelomic invagination. Therefore, Wnt9b may act upstream of Wnt7a in the caudal extension of the MD [29]. There is no clear evidence of a direct biological interaction between Wnt9b and Wnt7a, and further functional studies of the molecular mechanism underlying their interaction are required.

We explored the interactive effects among 6 relevant SNVs (from 17 loci, excluding 2 SNPs that were not in HWE, 7 loci without variants and 2 loci in linkage disequilibrium (LD)) using MDR software to test the hypothesis that the joint functioning of candidate genes causes an increased risk of MRKH syndrome. The ultimate goal of MDR analysis is hypothesis generation. Therefore, this approach may be preferred to reduce the risk of false negatives. We applied the non-parametric approach and found many three- and four-way gene-gene interactions in MRKH syndrome. The best four-way interaction model indicated by MDR analysis (Fig 2) supported the following novel and bold assumption that the dimensional interactions of AMH, PBX1, WNT7A and WNT9B may play a role in the etiology of MRKH syndrome during MD development. Some results of studies of the cellular mechanisms of MD formation and regression in mice are also in support of this hypothesis [6]. Regression of the MD is initiated by AMH signaling via Amhr2 [37]. WNT7a is a candidate signaling molecule that is secreted by the MD mesoepithelium to activate Amhr2 [36]. β-Catenin, which is a mediator of the canonical WNT pathway, functions upstream and downstream of AMH signaling during the process of Müllerian regression [6, 38, 39]. Wnt proteins act primarily through the canonical β-catenin-dependent pathway. Recently, a valuable gene expression profile of *in vitro* cultured vaginal tissue from MRKH syndrome patients has identified dysregulation of MUC1, WNT7A, JAG1 and DLL1, emphasizing the critical role of canonical Wnt signaling and the NOTCH pathway [40]. Therefore, it can be reasonably assumed that WNT9b and WNT7a, as mediated by β-catenin of the canonical WNT pathway, alter the mesenchymal signaling network, leading to changes in cell fate during Müllerian development through AMH signaling.

Notably, gene-gene interactions among Wnt9b, Wnt7a and Pbx1 were detected in additive and multiplicative models in this Chinese cohort. These results highlight the abnormal regulation of the WNT pathway in the pathogenesis of MRKH syndrome and especially indicate a critical role of the extracellular signaling factors Wnt9b. This hypothesis was already known, but our four-way interaction model may provide a theoretical basis for the molecular genetics of this syndrome and our findings suggest that MRKH syndrome is a complex polygenic disease. The studied WNT9B, WNT7A and PBX1 genes contributed small or moderate effects to the disease manifestation. However, none of them was sufficient to induce the MRKH phenotype. Variants of these genes might interfere with interactions or the balance among these genes, disturb the precise coordination of cellular programs during female reproductive tract development, and ultimately result in MRKH syndrome. However, further studies of the molecular mechanisms underlying MD development are required to elucidate how these genes actually interrelate and interact with each other.

## Conclusion

This study is the first to evaluate a series of candidate genes loci in a relatively large, but still limited, cohort of Chinese patients with MRKH syndrome. This study successfully identified two known SNPs (WNT9B and PBX1) that were separately and synergistically associated with MRKH syndrome and increased its risk in this case-control cohort. Notably, we found a novel dimensional epistatic four-gene effect (AMH, PBX1, WNT7A and WNT9B) in MRKH syndrome, providing novel data to contribute to the elucidation of the genetic mechanisms underlying its multifactorial etiology. In summary, our results suggest that a disordered network of developmental genes result in MRKH syndrome. However, the approach based on gene-gene epistatic interactions restricted the validity and assessment of the results of this study, and corresponding functional studies with suitable *in vitro* models are needed. Fine mapping and functional analyses of these newly identified associated loci may be conducted to confirm the causal variants and interactions, and deepen our knowledge of the underlying mechanisms of MRKH syndrome.

## Supporting Information

S1 Table Genotype frequencies of reported candidate loci. (DOC)

S2 Table Mean age of the patients and healthy controls studied. (DOC)

S3 Table Sex hormone and karyotype analysis. (DOC)

S4 Table Associated malformations. (DOC)

S5 Table Primer sequences of Sequenom MassARRAY system. (DOC)

S6 Table Allele characteristics of each candidate locus in Chinese Han. (DOC)

S7 Table Genetic model analysis of MRKH type I and type II. (XLSX)

S8 Table Additive interaction analysis of genes involved in MRKH syndrome in genotype combinations by chi-square test using 2×2 factorial design. (DOC)
