# Supplementary material for: Associations of Polymorphisms in WNT9B and PBX1 with Mayer-Rokitansky-Küster-Hauser Syndrome in Chinese Han
Source: PLoS One. 2015 Jun 15;10(6):e0130202. doi: 10.1371/journal.pone.0130202 (PMC4468103; doi:10.1371/journal.pone.0130202)
Supplement: S1 Table — (DOC) [file pone.0130202.s001.doc]

**Table1 S1 Genotype frequencies of reported candidate loci**

| Position /SNP | Phenotype | Population | Genotype frequency | | | | | | | | | | |
| --- | --- | --- | --- | --- | --- | --- | --- | --- | --- | --- | --- | --- | --- |
|  |  |  | Cases | | | | Control Females | | | | Controls ^a^(F+M) | | |
|  |  |  | AA (%) | AB (%) | | BB (%) | AA (%) | AB (%) | | BB (%) | AA (%) | AB(%) | BB (%) |
| WNT4 c.35T>C | MRKH with hyperandrogenism | EUR | 0  (0.0) | 1  (3.6) | | 27 (96.4) | 0  (0.0) | 0  (0.0) | | 100 (100.0) | NA | NA | NA |
| rs16826648 | MDAs | CHB | 0  (0.0) | 1  (0.5) | | 188 (99.5) | 0  (0.0) | 0  (0.0) | | 100 (100.0) | 0  (0.0) | 0  (0.0) | 379 (100.0) |
| WNT4 c.697G>A | MRKH with hyperandrogenism | EUR | 0  (0.0) | 1  (25) | | 3  (75.0) | NA | NA | | NA | NA | NA | NA |
| WNT4 c.483C>T | MRKH | EUR | 0  (0.0) | 1  (9.1) | | 10 (90.9) | NA | NA | | NA | NA | NA | NA |
| rs2275558 | MDAs | CHB | 58 (30.2) | 60 (31.3) | | 74 (38.5) | NA | NA | | NA | 12 (12.4) | 40 (41.2) | 45 (46.4) |
| WNT7A c.342C>T | MDAs | CHB | 0  (0.0) | 1  (0.5) | | 190 (99.5) | 0  (0.0) | 0  (0.0) | | 192 (100.0) | NA | NA | NA |
| rs3762719 | MDAs | CHB | 41 (21.5) | 98 (51.3) | | 52 (27.2) | 48  (25.0) | 88 (45.8) | | 56  (29.1) | 23 (23.7) | 51 (52.6) | 23 (23.7) |
| rs3749319 | MDAs | CHB | 13  (6.8) | 65  (34.0) | | 113 (59.2) | NA | NA | | NA | 7  (7.2) | 38 (39.2) | 52 (53.6) |
| WNT7A c.861G>A | MRKH | EUR | 0  (0.0) | 1  (9.1) | | 10 (90.9) | NA | NA | | NA | NA | NA | NA |
| HOXA10 c.170A>G | MDAs | CHB | 0  (0.0) | 1  (0.9) | | 108  (99.1) | 0  (0.0) | 0  (0.0) | | 100 (100.0) | NA | NA | NA |
| HOXA11 c.113C>G | MDAs | Greeks | 0  (0.0) | 1  (3.3) | | 29 (96.7) | 0  (0.0) | 0  (0.0) | | 100 (100.0) | NA | NA | NA |
| rs2070074 | MRKH | MXL | 0  (0.0) | 1  (6.7) | | 14 (93.3) | 0  (0.0) | 0  (0.0) | | 50 (100.0) | 0  (0.0) | 4  (6.1) | 62 (93.9) |
| LHX1 c.791G>C | MRKH | EUR | 0  (0.0) | 1  (1.8) | | 55 (98.2) | 0  (0.0) | 0  (0.0) | | 260 (100.0) | NA | NA | NA |
| rs4968281 | MRKH | CHS | 6  (14.3) | 25 (59.5) | | 11 (26.2) | 10  (23.8) | 20 (47.6) | | 12  (28.6) | 39 (19.8) | 106  (53.8) | 52 (26.4) |
| rs34072914 | MRKH | CHS | 1  (2.4) | 3  (7.1) | | 38 (90.5) | 0  (0.0) | 5  (11.9) | | 37  (88.1) | 0  (0.0) | 12  (6.1) | 185 (93.9) |
| WNT9B c.*158 C>T | MRKH | CHS | 0  (0.0) | 1  (2.4) | | 41 (97.6) | 0  (0.0) | 0  (0.0) | | 42 (100.0) | NA | NA | NA |
| AMH c.934C>T | MRKH | MXL | 20^b^  (66.7 ^b^) | | 10 ^b^  (33.3 ^b^) | | 34 ^b^  (68.0 ^b^) | | 16 ^b^  (32.0 ^b^) | | NA | NA | NA |

1. Genotype frequency of the 1000 Genomes release 15- Sep 2014
2. Allele frequency
