# Supplementary material for: Associations of Polymorphisms in WNT9B and PBX1 with Mayer-Rokitansky-Küster-Hauser Syndrome in Chinese Han
Source: PLoS One. 2015 Jun 15;10(6):e0130202. doi: 10.1371/journal.pone.0130202 (PMC4468103; doi:10.1371/journal.pone.0130202)
Supplement: S2 Table — (DOC) [file pone.0130202.s002.doc]

**Table S2. Mean age** of the patients and healthy controls studied.

|  | **MRKH** | **Healthy controls** |
| --- | --- | --- |
| n | 182 | 228 |
| Age | 25.30±5.02 | 30.56±6.03 |

Values were presented by n or mean±SD.
