# Supplementary material for: Associations of Polymorphisms in WNT9B and PBX1 with Mayer-Rokitansky-Küster-Hauser Syndrome in Chinese Han
Source: PLoS One. 2015 Jun 15;10(6):e0130202. doi: 10.1371/journal.pone.0130202 (PMC4468103; doi:10.1371/journal.pone.0130202)
Supplement: S3 Table — (DOC) [file pone.0130202.s003.doc]

**Table S3. Sex hormone and karyotype analysis**

|  | **MRKH** | Reference Range |
| --- | --- | --- |
| TSTE(ng/dl) | 43.81±20.57 | 14~76 |
| eE2(pg/ml) | 108.01±105.21 | 19~528 |
| FSH(mIU/ml) | 8.19±14.62 | 1.5~33.4 |
| LH(mIU/ml) | 9.25±11.83 | 0.5~76.3 |
| Karyotype | 46, XX | 46, XX |

Values were presented by mean±SD.
