# Supplementary material for: Associations of Polymorphisms in WNT9B and PBX1 with Mayer-Rokitansky-Küster-Hauser Syndrome in Chinese Han
Source: PLoS One. 2015 Jun 15;10(6):e0130202. doi: 10.1371/journal.pone.0130202 (PMC4468103; doi:10.1371/journal.pone.0130202)
Supplement: S4 Table — (DOC) [file pone.0130202.s004.doc]

**Table S4.** Associated malformations

| **TYPE** | **NUM.** |
| --- | --- |
| Isolated utero-vaginal aplasia | 155 |
| With renal malformation | 17 |
| With skeletal malformations | 4 |
| With hernia | 3 |
| With schizophrenia | 1 |
| With atrial septal defect | 1 |
| With renal and skeletal malformations | 1 |
