# Supplementary material for: Associations of Polymorphisms in WNT9B and PBX1 with Mayer-Rokitansky-Küster-Hauser Syndrome in Chinese Han
Source: PLoS One. 2015 Jun 15;10(6):e0130202. doi: 10.1371/journal.pone.0130202 (PMC4468103; doi:10.1371/journal.pone.0130202)
Supplement: S5 Table — (DOC) [file pone.0130202.s005.doc]

**Table S5. Primer sequences of Sequenom MassARRAY system.**

| **Gene variant** | **1^st^ PCR primer** | **2^nd^ PCR primer** | **Extend primer** |
| --- | --- | --- | --- |
| LHX1 c.791G>C | ACGTTGGATGAGAAGGAGAAGGGACCATTG | ACGTTGGATGACGCCTTCTTCCGCAGTCC | ccCGGCCGCTGGTGGACC |
| WNT4 c.35T>C | ACGTTGGATGACCATGAGTCCCCGCTCGT | ACGTTGGATGAGACTTACAGCCAGTTGCTC | gGACGGCGAAGACGAGGA |
| WNT4 rs16826648 | ACGTTGGATGAGGAGTGCCAGTACCAGTTC | ACGTTGGATGTCACCACCTTGCCGAAGAC | AGACGGGCAAGGAGTCA |
| WNT4 c.697G>A | ACGTTGGATGCATCCACAAACGACTGTGAG | ACGTTGGATGTTCCAGTGGTCAGGATGCTC | CTGACAACATCGCCTAC |
| WNT4 c.483C>T | ACGTTGGATGTGTGAGGTAAAGACGTGCTG | ACGTTGGATGTGGCACCATCAAACTTCTCC | gggcCCATCAAACTTCTCCTTCAGTGC |
| WNT9B rs4968281 | ACGTTGGATGTCCTCCCCACCTCTCTTGA | ACGTTGGATGAGTTTCAGTTCCGGCATGAG | gggcGCCTGGAGGGCAGGAC |
| WNT9B rs34072914 | ACGTTGGATGTTTCCTGTACGCGGTGTCCT | ACGTTGGATGGAGAGTCATCACAGGTGCAG | CCCAGCGCTGCAGGCC |
| WNT9B c.*158 C>T | ACGTTGGATGTGCATAAACCGGCATGTGTG | ACGTTGGATGTTGTTGAGTATCGAGGGAGG | cctGGAGGCAAAAGGCTGA |
| WNT7A rs3749319 | ACGTTGGATGAGGAGCTCAAAGTGGGTATG | ACGTTGGATGCTGAGGGTATTCTGGTGTCC | CTGGTGTCCCTAGAGCCG |
| WNT7A c.342C>T | ACGTTGGATGTCTTTGTCGCAGCCACAGTC | ACGTTGGATGGTTCACCTACGCCATCATTG | atcaaCTACGCCATCATTGCCGCCGGC |
| WNT7A rs3762719 | ACGTTGGATGAGAAGCAAGGCCAGTACCAC | ACGTTGGATGCATCCACAAAGACCTTGGCG | aGTAGCGGATGTCGGCA |
| WNT7A c.861G>A | ACGTTGGATGGGAGCCGTCTTGTTGCAGG | ACGTTGGATGCTGGTGTACATCGAGAAGTC | tggtACCCGGTGACCGGCAGTGTA |
| HOXA10 c.861G>A | ACGTTGGATGTCATCAGCTCGGGCAGAGG | ACGTTGGATGGCAGGTAGACCCCGCCGTG | gaAGGTAGACCCCGCCGTGGGCGTAGC |
| HOXA11 c.113C>G | ACGTTGGATGTAGGAGTATGTCATTGGGCG | ACGTTGGATGTACTTACTACGTCTCGGGTC | gcctgCTTTTCTGCCCCAGACCCC |
| PBX1 rs2275558 | ACGTTGGATGGCTGATGCATTCCCATGCTG | ACGTTGGATGCCCCATCCTGCAAGTGCTG | ggattCAAGTGCTGGGACAGGCC |
| AMH c.934C>T | ACGTTGGATGTTCCTGGAGACGCTCACGC | ACGTTGGATGCCAGCGCGTCCGGATCCAG | aataCGGATCCAGGGCCAGGCA |
| GALT rs2070074 | ACGTTGGATGGGGTAGTAATGAGCGTGCAG | ACGTTGGATGCTCTCTTCTTTCTGTCAGGG | gaGCTGGGGCCAACTGGA |
