# Supplementary material for: Associations of Polymorphisms in WNT9B and PBX1 with Mayer-Rokitansky-Küster-Hauser Syndrome in Chinese Han
Source: PLoS One. 2015 Jun 15;10(6):e0130202. doi: 10.1371/journal.pone.0130202 (PMC4468103; doi:10.1371/journal.pone.0130202)
Supplement: S6 Table — (DOC) [file pone.0130202.s006.doc]

**Table S6.** Allele characteristics of each candidate locus in Chinese Han.

| Gene | SNP | HWE |  | |  | | Genotype frequency | | |  | | |  | | Allele frequency | | |
| --- | --- | --- | --- | --- | --- | --- | --- | --- | --- | --- | --- | --- | --- | --- | --- | --- | --- |
|  |  |  |  | MRKH | | Type I | | Type II | Controls | |  | MRKH | | Type I | | Type II | Controls |
| **PBX1**  Exon1 | rs2275558  c.61G>A  Ns p.G21S |  | AA | 66(36.3) | | 54(34.8) | | 12(44.4) | 84(36.8) | | A | 226(62.1) | | 190(61.3) | | 36(66.7) | 267(58.8) |
|  |  | 0.280 | AG | 94(51.6) | | 82(52.9) | | 12(44.4) | 99(43.6) | | G | 138(37.9) | | 120(38.7) | | 18(33.3) | 187(41.2) |
|  |  |  | GG | 22(12.1) | | 19(12.3) | | 3(11.1) | 44(19.4) | |  |  | |  | |  |  |
| **WNT4** | Novel |  | CC | 0 | | 0 | | 0 | 3(1.3) | | C | 0 | | 0 | | 0 | 9(2.0) |
| Exon1 | c.35T>C | <0.01 | CT | 0 | | 0 | | 0 | 3(1.3) | | T | 358 | | 306 | | 54 | 445(98.0) |
|  | Ns p.L12P |  | TT | 179 | | 153 | | 27 | 221(97.4) | |  |  | |  | |  |  |
| **WNT4** | rs16826648 |  | AA | 0 | | 0 | | 0 | 0 | | A | 0 | | 0 | | 0 | 0 |
| Exon2 | c.276G>A | NA | AG | 0 | | 0 | | 0 | 0 | | G | 364 | | 310 | | 54 | 456 |
|  | Sy p.L92L |  | GG | 182 | | 155 | | 27 | 228 | |  |  | |  | |  |  |
| **WNT4** | Novel |  | AA | 0 | | 0 | | 0 | 0 | | A | 0 | | 0 | | 0 | 0 |
| Exon3 | c.697G>A | NA | AG | 0 | | 0 | | 0 | 0 | | G | 364 | | 310 | | 54 | 456 |
|  | Ns p.A233T |  | GG | 182 | | 155 | | 27 | 228 | |  |  | |  | |  |  |
| **WNT4** | Novel |  | TT | 0 | | 0 | | 0 | 0 | | T | 0 | | 0 | | 0 | 0 |
| Exon3 | c.483C>T | NA | TC | 0 | | 0 | | 0 | 0 | | C | 364 | | 310 | | 54 | 456 |
|  | Sy p.Y161Y |  | CC | 182 | | 155 | | 27 | 228 | |  |  | |  | |  |  |
| **WNT7A** | rs3749319 |  | AA | 104(57.1) | | 90(58.1) | | 14(51.9) | 139(60.9) | | A | 272(74.7) | | 233(75.2) | | 39(72.2) | 352(77.2) |
| Intron2 | c.298+37C>A | 0.240 | AC | 64(35.2) | | 53(34.2) | | 11(40.7) | 74(32.5) | | C | 92(25.3) | | 77(24.8) | | 15(27.8) | 104(2.8) |
|  |  |  | CC | 14(7.7) | | 12(7.7) | | 2(7.4) | 15(6.6) | |  |  | |  | |  |  |
| **WNT7A** | Novel |  | TT | 0 | | 0 | | 0 | 0 | | T | 0 | | 0 | | 0 | 0 |
| Exon3 | c.342C>T | NA | TC | 0 | | 0 | | 0 | 0 | | C | 364 | | 310 | | 54 | 456 |
|  | Sy p.G114G |  | CC | 182 | | 155 | | 27 | 228 | |  |  | |  | |  |  |
| **WNT7A** | rs3762719 |  | CC | 51(28.0) | | 38(24.5) | | 13(48.1) | 72(31.6) | | C | 187(51.4) | | 154(49.7) | | 33(61.1) | 250(54.8) |
| Exon3 | c.459T>C | 0.382 | CT | 85(46.7) | | 78(50.3) | | 7(25.9) | 106(46.5) | | T | 177(48.6) | | 156(50.3) | | 21(38.9) | 206(45.2) |
|  | Sy p.S153S |  | TT | 46(25.3) | | 39(25.2) | | 7(25.9) | 50(21.9) | |  |  | |  | |  |  |
| **WNT7A** | Novel |  | AA | 0 | | 0 | | 0 | 0 | | A | 0 | | 0 | | 0 | 0 |
| Exon4 | c.861G>A | NA | AG | 0 | | 0 | | 0 | 0 | | G | 364 | | 310 | | 54 | 456 |
|  | Sy p.287V>V |  | GG | 182 | | 155 | | 27 | 228 | |  |  | |  | |  |  |
| **HOXA10** | Novel |  | GG | 0(0.0) | | 0(0.0) | | 0(0.0) | 0(0.0) | | G | 1(0.3) | | 0(0.0) | | 1(1.9) | 3(0.7) |
| Exon2 | Ns c.170A>G | 0.946 | GA | 1(0.6) | | 0(0.0) | | 1(3.8) | 3(1.3) | | A | 357(99.7) | | 306(100.0) | | 51(98.1) | 445(99.3) |
|  | p. Y57C |  | AA | 178(99.4) | | 153 | | 25(96.2) | 221(98.7) | |  |  | |  | |  |  |
| **HOXA11** | Novel |  | GG | 0 | | 0 | | 0 | 0 | | G | 0 | | 0 | | 0 | 0 |
| Exon1 | c.113C>G | NA | GC | 0 | | 0 | | 0 | 0 | | C | 364 | | 310 | | 54 | 456 |
|  | Ns p.P38R |  | CC | 182 | | 155 | | 27 | 228 | |  |  | |  | |  |  |
| **GALT** | rs2070074 |  | GG | 0(0.0) | | 0(0.0) | | 0(0.0) | 0(0.0) | | G | 3(0.8) | | 3(1.0) | | 0(0.0) | 4(0.9) |
| Exon10 | c.940A>G | 0.867 | GA | 3(1.6) | | 3(1.9) | | 0(0.0) | 4(1.8) | | A | 361(99.2) | | 307(99.0) | | 54(100.0) | 452(99.1) |
|  | Ns p.N314D |  | AA | 179(98.4) | | 152(98.1) | | 27(100.0) | 224(98.2) | |  |  | |  | |  |  |
| **LHX1** | Novel |  | CC | 182 | | 155 | | 27 | 228 | | C | 364 | | 310 | | 54 | 456 |
| Exon4 | c.791G>C | NA | CG | 0 | | 0 | | 0 | 0 | | G | 0 | | 0 | | 0 | 0 |
|  | Ns p.R264P |  | GG | 0 | | 0 | | 0 | 0 | |  |  | |  | |  |  |
| **WNT9B** | rs4968281 |  | CC | 42(23.1) | | 35(22.6) | | 7(25.9) | 66(28.9) | | C | 162(44.5) | | 138(44.5) | | 24(44.4) | 227(49.8) |
| Exon2 | c.317T>C | 0.002 | CT | 78(42.8) | | 68(43.9) | | 10(37) | 95(41.7) | | T | 202(55.5) | | 172(55.5) | | 30(55.6) | 229(50.2) |
|  | Ns p.M106T |  | TT | 62(34.1) | | 52(33.5) | | 10(37) | 67(29.4) | |  |  | |  | |  |  |
| **WNT9B** | rs34072914 |  | TT | 1(0.5) | | 1(0.6) | | 0(0.0) | 0(0.0) | | T | 16(4.4) | | 12(3.9) | | 4(7.4) | 8(1.8) |
| Exon3 | c.399G>T | 0.761 | TG | 14(7.7) | | 10(6.5) | | 4(14.8) | 8(3.5) | | G | 348(95.6) | | 298(96.1) | | 50(92.6) | 448(98.2) |
|  | Sy p.R133R |  | GG | 167(91.8) | | 144(92.2) | | 23(85.2) | 220(96.5) | |  |  | |  | |  |  |
| **WNT9B** | Novel |  | TT | 0(0.0) | | 0(0.0) | | 0(0.0) | 0(0.0) | | T | 1(0.3) | | 1(0.3) | | 0(0.0) | 0(0.0) |
| Exon4 | c.*158 C>T | 1.000 | TC | 1(0.5) | | 1(0.6) | | 0(0.0) | 0(0.0) | | C | 363(99.7) | | 309(99.7) | | 54(100) | 456(100) |
|  | UTR3 |  | CC | 181(99.5) | | 154(99.4) | | 27(100) | 228(100) | |  |  | |  | |  |  |
| **AMH** | Novel |  | TT | 161(94.2) | | 136(93.8) | | 25(96.2) | 223(97.8) | | T | 332(97.1) | | 281(96.9) | | 51(98.1) | 451(98.9) |
| Exon5 | c.934C>T | 0.836 | TC | 10(5.8) | | 9(6.2) | | 1(3.8) | 5(2.2) | | C | 10(2.9) | | 9(3.1) | | 1(1.9) | 5(1.1) |
|  | Ns p.R312C |  | CC | 0(0.0) | | 0(0.0) | | 0(0.0) | 0(0.0) | |  |  | |  | |  |  |
