# Supplementary material for: Associations of Polymorphisms in WNT9B and PBX1 with Mayer-Rokitansky-Küster-Hauser Syndrome in Chinese Han
Source: PLoS One. 2015 Jun 15;10(6):e0130202. doi: 10.1371/journal.pone.0130202 (PMC4468103; doi:10.1371/journal.pone.0130202)
Supplement: S8 Table — (DOC) [file pone.0130202.s008.doc]

**Table S7** Additive interaction analysis of genes involved in MRKH syndrome in genotype combinations by chi-square test using 2×2 factorial design

| **Combinations** | **MRKH** | **Controls** | ***p _vs. con_*** | **OR (95% CI)** |
| --- | --- | --- | --- | --- |
| *PBX1(rs2275558)*/*WNT7A(rs3749319)*-0/0 | 2 | 3 | 1.00 | 0.830(0.137-5.019) |
| *PBX1(rs2275558)*/*WNT7A(rs3749319)*-0/1 | 20 | 41 | 0.046 | 0.560(0.315-0.995) |
| *PBX1(rs2275558)*/*WNT7A(rs3749319)*-1/0 | 12 | 12 | 0.576 | 1.265(0.554-2.886) |
| *PBX1(rs2275558)*/*WNT7A(rs3749319)*-1/1 | 148 | 171 | 0.146 | 1.426(0.882-2.303) |
| *PBX1(rs2275558)*/*WNT7A(rs3762719)*-0/0 | 7 | 11 | 0.624 | 0.785(0.298-2.069) |
| *PBX1(rs2275558)*/*WNT7A(rs3762719)*-0/1 | 15 | 33 | 0.049 | 0.528(0.27-1.006) |
| *PBX1(rs2275558)*/*WNT7A(rs3762719)*-1/0 | 39 | 39 | 0.277 | 1.315(0.802-2.155) |
| *PBX1(rs2275558)*/*WNT7A(rs3762719)*-1/1 | 121 | 144 | 0.521 | 1.143(0.759-1.722) |
| *PBX1(rs2275558)*/*HOXA10(c.A170G)*-0/0 | 22 | 43 | 0.058 | 0.587(0.336-1.023) |
| *PBX1(rs2275558)*/*HOXA10(c.A170G)*-0/1 | 0 | 0 |  |  |
| *PBX1(rs2275558)*/*HOXA10(c.A170G)*-1/0 | 156 | 177 | 0.040 | 1.763(1.022-3.040) |
| *PBX1(rs2275558)*/*HOXA10(c.A170G)*-1/1 | 1 | 3 | 0.632 | 0.412(0.042-3.995) |
| *PBX1(rs2275558)*/*GALT(rs2070074)*-0/0 | 21 | 44 | 0.031 | 0.542(0.309-0.951) |
| *PBX1(rs2275558)*/*GALT(rs2070074)*-0/1 | 1 | 0 | 0.445 |  |
| *PBX1(rs2275558)*/*GALT(rs2070074)*-1/0 | 158 | 179 | 0.036 | 1.765(1.034-3.013) |
| *PBX1(rs2275558)*/*GALT(rs2070074)*-1/1 | 2 | 4 | 0.697 | 0.619(0.112-3.420) |
| *PBX1(rs2275558)*/*WNT9B*(rs34072914*)*-0/0 | 20 | 42 | 0.035 | 0.544(0.307-0.964) |
| *PBX1(rs2275558)*/*WNT9B*(rs34072914*)*-0/1 | 2 | 2 | 1.000 | 1.250(0.174-8.961) |
| *PBX1(rs2275558)*/*WNT9B*(rs34072914*)*-1/0 | 147 | 177 | 0.489 | 1.186(0.731-1.925) |
| ***PBX1(rs2275558)*/*WNT9B*(rs34072914*)*-1/1** | 13 | 6 | **0.032** | **2.833(1.055-7.609)** |
| *PBX1(rs2275558)*/*AMH* (c.C934T*)*-0/0 | 0 | 0 |  |  |
| *PBX1(rs2275558)*/*AMH* (c.C934T*)*-0/1 | 19 | 44 | 0.025 | 0.520(0.291-0.928) |
| *PBX1(rs2275558)*/*AMH* (c.C934T*)*-1/0 | 0 | 0 |  |  |
| ***PBX1(rs2275558)*/*AMH* (c.C934T*)*-1/1** | 152 | 183 | **0.025** | **1.923(1.078-3.434)** |
| *WNT7A(rs3749319)*/ *WNT7A(rs3762719)*-0/0 | 12 | 11 | 0.439 | 1.393(0.600-3.233) |
| *WNT7A(rs3749319)*/ *WNT7A(rs3762719)*-0/1 | 2 | 4 | 0.697 | 0.622(0.113-3.436) |
| *WNT7A(rs3749319)*/ *WNT7A(rs3762719)*-1/0 | 34 | 39 | 0.679 | 1.113(0.670-1.850) |
| *WNT7A(rs3749319)*/ *WNT7A(rs3762719)*-1/1 | 134 | 174 | 0.531 | 0.866(0.553-1.358) |
| *WNT7A(rs3749319)*/ *HOXA10(c.A170G)*-0/0 | 44 | 48 | 0.454 | 1.195(0.750-1.905) |
| *WNT7A(rs3749319)*/ *HOXA10(c.A170G)*-0/1 | 1 | 1 | 1.000 | 1.253(0.078-20.170) |
| *WNT7A(rs3749319)*/ *HOXA10(c.A170G)*-1/0 | 134 | 173 | 0.579 | 0.878(0.554-1.391) |
| *WNT7A(rs3749319)*/ *HOXA10(c.A170G)*-1/1 | 0 | 2 | 0.505 | - |
| *WNT7A(rs3749319)*/ *GALT(rs2070074)*-0/0 | 45 | 48 | 0.378 | 1.232(0.775-1.958) |
| *WNT7A(rs3749319)*/ *GALT(rs2070074)*-0/1 | 1 | 2 | 1.000 | 0.624(0.056-6.940) |
| *WNT7A(rs3749319)*/ *GALT(rs2070074)*-1/0 | 134 | 176 | 0.403 | 0.825(0.525-1.296) |
| *WNT7A(rs3749319)*/ *GALT(rs2070074)*-1/1 | 2 | 2 | 1.000 | 1.256(0.175-9.001) |
| *WNT7A(rs3749319)*/ *WNT9B*(rs34072914*)*-0/0 | 43 | 47 | 0.464 | 1.191(0.745-1.904) |
| *WNT7A(rs3749319)*/ *WNT9B*(rs34072914*)*-0/1 | 3 | 3 | 1.000 | 1.257(0.251-6.303) |
| *WNT7A(rs3749319)*/ *WNT9B*(rs34072914*)*-1/0 | 124 | 173 | 0.081 | 0.680(0.440-1.050) |
| ***WNT7A(rs3749319)*/ *WNT9B*(rs34072914*)*-1/1** | 12 | 5 | **0.026** | **3.148(1.088-9.107)** |
| *WNT7A(rs3749319)*/ *AMH* (c.C934T*)*-0/0 | 0 | 0 |  |  |
| *WNT7A(rs3749319)*/ *AMH* (c.C934T*)*-0/1 | 44 | 50 | 0.376 | 1.233(0.775-1.963) |
| *WNT7A(rs3749319)*/ *AMH* (c.C934T*)*-1/0 | 0 | 0 |  |  |
| *WNT7A(rs3749319)*/ *AMH* (c.C934T*)*-1/1 | 127 | 178 | 0.376 | 0.811(0.509-1.290) |
| *WNT7A(rs3762719)*/ *HOXA10(c.A170G)*-0/0 | 44 | 48 | 0.454 | 1.195(0.750-1.905) |
| *WNT7A(rs3762719)*/ *HOXA10(c.A170G)*-0/1 | 1 | 1 | 1.000 | 1.253(0.078-20.170) |
| *WNT7A(rs3762719)*/ *HOXA10(c.A170G)*-1/0 | 134 | 173 | 0.579 | 0.878(0.554-1.391) |
| *WNT7A(rs3762719)*/ *HOXA10(c.A170G)*-1/1 | 0 | 2 | 0.505 | - |
| *WNT7A(rs3762719)*/ *GALT(rs2070074)*-0/0 | 45 | 48 | 0.378 | 1.232(0.775-1.958) |
| *WNT7A(rs3762719)*/ *GALT(rs2070074)*-0/1 | 1 | 2 | 1.000 | 0.624(0.056-6.940) |
| *WNT7A(rs3762719)*/ *GALT(rs2070074)*-1/0 | 134 | 176 | 0.403 | 0.825(0.525-1.296) |
| *WNT7A(rs3762719)*/ *GALT(rs2070074)*-1/1 | 2 | 2 | 1.000 | 1.256(0.175-9.001) |
| *WNT7A(rs3762719)*/ *WNT9B*(rs34072914*)*-0/0 | 43 | 47 | 0.464 | 1.191(0.745-1.904) |
| *WNT7A(rs3762719)*/ *WNT9B*(rs34072914*)*-0/1 | 3 | 3 | 1.000 | 1.257(0.251-6.303) |
| *WNT7A(rs3762719)*/ *WNT9B*(rs34072914*)*-1/0 | 124 | 173 | 0.081 | 0.680(0.440-1.050) |
| ***WNT7A(rs3762719)*/ *WNT9B*(rs34072914*)*-1/1** | 12 | 5 | **0.026** | **3.148(1.088-9.107)** |
| *WNT7A(rs3762719)*/ *AMH* (c.C934T*)*-0/0 | 0 | 0 |  |  |
| *WNT7A(rs3762719)*/ *AMH* (c.C934T*)*-0/1 | 44 | 50 | 0.376 | 1.233(0.775-1.963) |
| *WNT7A(rs3762719)*/ *AMH* (c.C934T*)*-1/0 | 0 | 0 |  |  |
| *WNT7A(rs3762719)*/ *AMH* (c.C934T*)*-1/1 | 127 | 178 | 0.376 | 0.811(0.509-1.290) |
| *HOXA10(c.A170G)* / *GALT(rs2070074)*-0/0 | 175 | 218 | 1.000 | 1.204(0.335-4.334) |
| *HOXA10(c.A170G)* / *GALT(rs2070074)*-0/1 | 3 | 3 | 1.000 | 1.256(0.250-6.298) |
| *HOXA10(c.A170G)* / *GALT(rs2070074)*-1/0 | 1 | 2 | 1.000 | 0.624(0.056-6.933) |
| *HOXA10(c.A170G)* / *GALT(rs2070074)*-1/1 | 0 | 1 | 1.000 | 1.004(0.996-1.013) |
| *HOXA10(c.A170G)* / *WNT9B*(rs34072914*)*-0/0 | 163 | 213 | 0.108 | 0.526(0.238-1.164) |
| *HOXA10(c.A170G)* / *WNT9B*(rs34072914*)*-0/1 | 15 | 8 | **0.039** | **2.470(1.023-5.964)** |
| *HOXA10(c.A170G)* / *WNT9B*(rs34072914*)*-1/0 | 1 | 3 | 0.633 | 0.414(0.043-4.013) |
| *HOXA10(c.A170G)* / *WNT9B*(rs34072914*)*-1/1 | 0 | 0 |  |  |
| *HOXA10(c.A170G)* / *AMH* (c.C934T*)*-0/0 | 0 | 0 |  |  |
| *HOXA10(c.A170G)* / *AMH* (c.C934T*)*-0/1 | 167 | 221 | 0.638 | 2.267(0.234-21.988) |
| *HOXA10(c.A170G)* / *AMH* (c.C934T*)*-1/0 | 0 | 0 |  |  |
| *HOXA10(c.A170G)* / *AMH* (c.C934T*)*-1/1 | 1 | 3 | 0.638 | 0.441(0.045-4.279) |
| *GALT(rs2070074)* / *WNT9B*(rs34072914*)* -0/0 | 164 | 216 | 0.074 | 0.506(0.237-1.080) |
| *GALT(rs2070074)* / *WNT9B*(rs34072914*)* -0/1 | 15 | 8 | **0.039** | **2.470(1.023-5.963)** |
| *GALT(rs2070074)* / *WNT9B*(rs34072914*)* -1/0 | 3 | 4 | 1.000 | 0.939(0.207-4.248) |
| *GALT(rs2070074)* / *WNT9B*(rs34072914*)* -1/1 | 0 | 0 |  |  |
| *GALT(rs2070074)* / *AMH* (c.C934T*)*-0/0 | 0 | 0 |  |  |
| *GALT(rs2070074)* / *AMH* (c.C934T*)*-0/0 | 169 | 224 | 0.704 | 1.509(0.273-8.335) |
| *GALT(rs2070074)* / *AMH* (c.C934T*)*-0/0 | 0 | 0 |  |  |
| *GALT(rs2070074)* / *AMH* (c.C934T*)*-0/0 | 2 | 4 | 0.704 | 0.663(0.120-3.661) |
| *WNT9B*(rs34072914*)* / *AMH* (c.C934T*)*-0/0 | 0 | 0 |  |  |
| *WNT9B*(rs34072914*)* / *AMH* (c.C934T*)*-0/1 | 157 | 220 | 0.043 | 0.408(0.167-0.995) |
| *WNT9B*(rs34072914*)* / *AMH* (c.C934T*)*-1/0 | 0 | 0 |  |  |
| ***WNT9B*(rs34072914*)* / *AMH* (c.C934T*)*-1/1** | 14 | 8 | **0.043** | **2.452(1.005-5.986)** |

Genotype combinations were conducted under the dominant model.

*PBX1*(rs2275558) 0/1: GG/GA+AA; *WNT7A*(rs3749319) 0/1: CC/CA+AA; *WNT7A*(rs3762719) 0/1: TT/TC+CC; *HOXA10*(c.A170G) 0/1: AA/AG+GG; *GALT*(rs2070074) 0/1: AA/AG+GG; *WNT9B*(rs34072914) 0/1: GG/GT+TT; *AMH*(c.C934T) 0/1: CC/CT+TT.

Interactions were conducted by direct counting and chi-square tests using a 2×2 factorial design to calculate the attributable proportion due to interaction (AP) and the relative excess risk due to interaction (RERI).
